# Toll-like Receptor Activation Remodels the Polyamine and Tryptophan Metabolism in Porcine Macrophages

**DOI:** 10.3390/metabo15030162

**Published:** 2025-03-01

**Authors:** Meimei Zhang, Lingfei Du, Yinhao Shen, Peng Bin

**Affiliations:** 1 State Key Laboratory of Swine and Poultry Breeding Industry, College of Animal Science, South China Agricultural University, Guangzhou 510642, China; mmzhang23@scau.edu.cn (M.Z.); lingfeidu0329@163.com (L.D.); shenyinhao@stu.scau.edu.cn (Y.S.); 2 Henry Fok School of Biology and Agriculture, Shaoguan University, Shaoguan 512005, China

**Keywords:** Macrophage, TLRs, polyamine, tryptophan

## Abstract

**Background:** The early nutritional metabolism of piglets is intimately associated with the regulation of immune function, and amino acids play a crucial role in modulating the fate and function of porcine immune cells, especially macrophages. However, the metabolic changes upon macrophage activation remain elusive. **Methods:** We established an in vitro activation model of porcine macrophages and investigated alterations in metabolites involved in polyamine and tryptophan metabolism upon activation by various toll-like receptor (TLR) activators. **Results:** TLR activation inhibits the production of spermine and alters the kynurenine pathway of the tryptophan metabolism toward the kynurenic acid biosynthesis. Specifically, TLR9 activation redirects the metabolic pathway of tryptophan toward kynurenic acid synthesis, which subsequently inhibits melatonin production via the protein kinase A (PKA)/cyclic adenosine monophosphate (cAMP)/cAMP-responsive element-binding protein (CREB) signaling pathways. **Conclusions:** TLR activation reprograms the polyamine and tryptophan metabolism in porcine macrophages. Knowledge of the metabolic alterations in polyamine and tryptophan upon TLR activation in macrophages offers valuable insights and potential strategies for nutritional intervention to enhance piglet immunity.

## 1. Introduction

To further improve the reproductive performance of sows in swine husbandry, early weaning of piglets is implemented. This practice reduces the farrowing interval and increases the annual productivity of sows. However, early weaned piglets are particularly susceptible to environmental stressors, such as pathogens, due to their immature immune systems. This predisposition leads to enteric inflammation and subsequent diarrhea [1]. Macrophages, among the earliest immune cells to develop in neonatal piglets, play a crucial role in the early immune response of weaned piglets, particularly in combating pathogen infection [2]. Metabolic alterations, particularly in the amino acid metabolism, frequently govern the phenotypes of macrophages by regulating transcription and post-transcriptional processes. For instance, γ-amino butyric acid (GABA) activates the succinate–flavin adenine dinucleotide (FAD)–lysine-specific demethylase1 (LSD1) signaling pathway and regulates the histone demethylation of *Bcl2l11* and *Dusp2*, thereby orchestrating macrophage maturation and inflammation [3]. Therefore, modulating the amino acid metabolism of macrophages has emerged as a promising strategy to shape the functionality of the intestinal immune system in weaned piglets.

Toll-like receptors (TLRs) recognize various pathogen-associated molecular patterns (PAMPs), such as lipopolysaccharide (LPS), polyinosinic–polycytidylic acid (poly I:C), and peptidoglycan (PGN). These PAMPs trigger intracellular signal transduction cascades, which induce inflammatory responses in macrophages [4]. Furthermore, TLR activation reprograms the intracellular metabolism and enhances the effector functions of macrophages [5]. For example, TLR4-activated macrophages exhibit a transient increase in aerobic glycolysis rates. This metabolic state promotes the expression of LPS-responsive gene sets by upregulation of ATP-citrate lyase activity and histone acetylation [6]. Therefore, understanding the metabolic adaptations induced by TLR activation may provide novel insights into how changes in substrate accessibility affect gene expression.

Tryptophan, an essential amino acid, is implicated in the regulation of numerous biological processes, including protein biosynthesis, neurotransmission, and immune response [7]. It can be catabolized into indole derivatives, melatonin, and kynurenine through microbial and host pathways [8]. Tryptophan and its metabolites play a pivotal role in modulating macrophage functions. Our previous study demonstrated that melatonin suppresses the production of IL-1β by transcriptionally downregulating *Ifngr2* and inhibiting the JAK-STAT pathway [9,10]. Consequently, investigating the alterations in the tryptophan metabolism upon macrophage activation is of great significance. Polyamines, polycations widely present in eukaryotic cells, play a crucial role in driving macrophage polarization [11,12]. There exists a profound interrelationship between tryptophan catabolism and polyamine metabolism, with spermine enhancing the mRNA expression of indoleamine 2,3-dioxygenase 1 (IDO1) via the nuclear factor–kappa B (NF-κB) signaling pathway [13,14]. A concurrent analysis of the tryptophan and polyamine metabolism will contribute to a more comprehensive understanding of their regulatory roles in macrophage function.

In this study, we discovered that the activation of TLR9 in porcine macrophages redirects the metabolic pathway of tryptophan toward kynurenic acid synthesis. This process potentially inhibits melatonin production via the protein kinase A (PKA)/cyclic adenosine monophosphate (cAMP)/cAMP-responsive element-binding protein (CREB) signaling pathway.

## 2. Materials and Methods

### 2.1. Cell Lines and Cell Culture

Porcine alveolar macrophages 3D4/21 cells were kindly donated by Prof. Huahua Du from Zhejiang University, and ANA.1 murine macrophages were kindly donated by Prof. Yuexia Liao from Yangzhou University. Cells were cultured at 37 °C and 5% CO_2_ in RPMI 1640 (Gibco, Waltham, MA, USA) medium supplemented with 10% fetal bovine serum (ExCell Bio, Shanghai, China) and 1% penicillin–streptomycin (Gibco, Waltham, MA, USA). Cells were polarized into proinflammatory subtypes by stimulating with lipopolysaccharide (1 mg/mL, Sigma, St. Louis, MO, USA) for 12 h. Both cells and supernatant were collected for further analysis. Each experiment was conducted using cells from different passages to ensure the robustness and reproducibility of our results.

### 2.2. Quantitative Real-Time Polymerase Chain Reaction (qRT-PCR)

Total RNA was extracted from the cells using the EZ-press RNA Purification Kit (EZ Bioscience, Roseville, MN, USA), following the manufacturer’s instructions. The quality and concentration of the RNA were assessed spectrophotometrically at 260 nm using the NanoDrop One spectrophotometer (Thermo Fisher Scientific, Waltham, MA, USA). Subsequently, 1 μg of diluted RNA was reverse-transcribed into cDNA using the Color Reverse Transcription Kit (EZ Bioscience). A quantitative real-time polymerase chain reaction (qPCR) was performed using the QuantStudio 6Pro (Thermo Fisher Scientific) and SYBR Green qPCR Master Mix (EZ Bioscience). The primer sequences used for the qPCR were presented in Table 1. The qPCR results were normalized against the internal gene *β-actin* and determined by the comparative CT (2^−ΔΔCT^) method.

### 2.3. Enzyme Linked Immunosorbent Assay (ELISA)

The intracellular levels of cyclic adenosine monophosphate (cAMP), arylalkylamine *N*-acetyltransferase (AANAT), and melatonin were measured using ELISA Kits (mlbio). The cells were harvested and lysed by repeated freezing and thawing, and the resulting cell lysates were analyzed following the manufacturer’s instructions.

### 2.4. Immunoblotting

For protein expression analysis, 1 × 10^6^ cells were harvested and lysed in RIPA Lysis Buffer (Beyotime Biotechnology, Beijing, China). Following sonication, the total protein concentration was determined using the Enhanced BCA Protein Assay Kit (Beyotime Biotechnology). The protein samples were denatured by heating at 95 °C for 5 min, and then separated using SDS-PAGE. The separated proteins were transferred onto polyvinylidene difluoride membranes. The membranes were blocked in 5% (*v*/*v*) milk for 90 min, incubated with primary antibodies overnight at 4 °C, and then treated with HRP secondary antibodies at room temperature for 90 min. A chemiluminescent reagent was introduced to the membrane, emitting light that was captured by photographic film in direct contact. Subsequent film development transformed the latent image into a visible one. The developed blots were analyzed using QuantityOne software (V4.6.6).

### 2.5. Metabolomic Analysis

The metabolites were measured using liquid chromatography–mass spectrometry (LC-MS). To prepare the samples, cellular lysate or supernatant was collected and centrifugated at 1000 rpm for 5 min to remove cellular debris. Subsequently, the supernatant was transferred to new tubes, and 800 μL methanol–acetonitrile solution (50:50, *v*/*v*) was added. The mixture was sonicated in ice water for 10 min and then centrifugated at 14,500 rpm for 15 min. The supernatant was vacuum-dried at 60 °C for 2 h and further dried with nitrogen to obtain a dry residue. This residue was redissolved in a 200 μL solution of methanol and water (50:50, *v*/*v*) and sonicated in ice water for another 10 min. Following centrifugation, the supernatant was filtered through a 0.22 μm membrane before being subjected to LC-MS analysis. The analytical parameters and conditions of the LC-MS analysis were based on our previous study [15].

### 2.6. Statistical Analysis

The statistical analyses presented in this study were performed using GraphPad Prism software (V8.0). The data were shown as the mean ± standard deviation (SD) or standard error of the mean (SEM). The data from the control and different TLR-activated groups were analyzed by one-way ANOVA followed by Dunnett’s multiple range test. The data between the two groups were determined using an unpaired *t* test when the data complied with Gaussian distribution and exhibited equal variance. In cases where the data complied with Gaussian distribution but had unequal variance, an unpaired *t* test with Welch’s correction was employed. Alternatively, non-parametric tests were used for data that deviated from a normal distribution. The differences were considered significant at *p* < 0.05.

## 3. Results

### 3.1. TLR Activation Remodeled the Polyamine Metabolism of Porcine Macrophages

**Figure 1:**
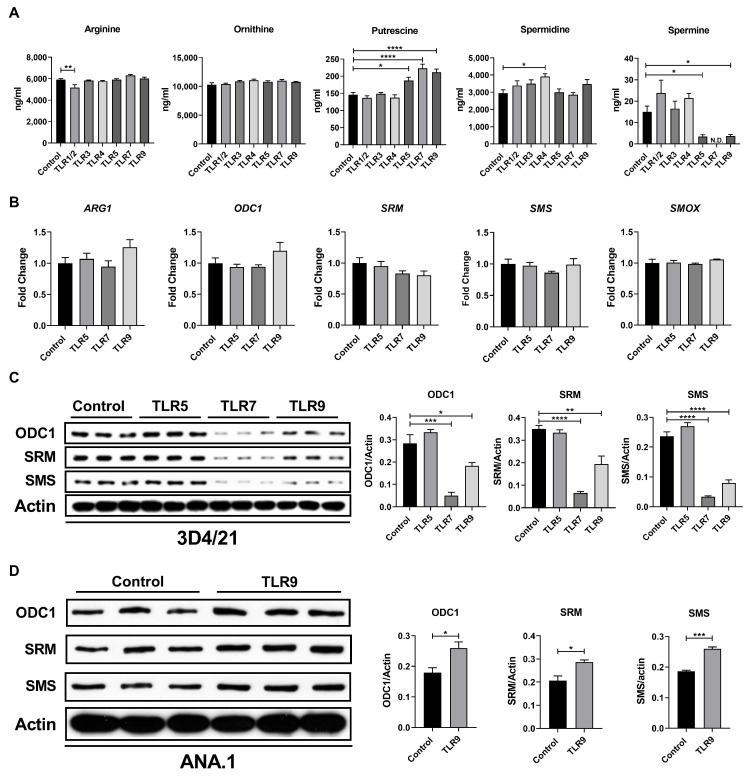
TLR activation remodels the polyamine metabolism in porcine macrophages. (**A**) Levels of polyamine metabolites in control or TLR-activated macrophages (*n* = 6). (**B**) Relative mRNA expressions of polyamine metabolism-related genes in control or TLR-activated macrophages (*n* = 5). (**C**) Protein expressions of ODC1, SRM, and SMS in control or TLR-activated 3D4/21 cells (*n* = 3). (**D**) Protein expressions of ODC1, SRM, and SMS in control or TLR9-activated ANA.1 cells (*n* = 3). Data were analyzed by an unpaired *t* test and exhibited as the mean ± SEM. * *p* < 0.05; ** *p* < 0.01; *** *p* < 0.001, **** *p* < 0.0001. ARG1, arginase 1; ODC1, ornithine decarboxylase; SRM, spermidine synthetase; SMS, spermine synthase; SMOX, spermine oxidase.

Given that spermine biosynthesis influences the tryptophan metabolism, we first investigated the metabolic alteration in polyamines in porcine macrophages after TLR activation. We stimulated 3D4/21 cells with various TLR activators [Pam3CSK4 for TLR1/2, Poly(I:C) for TLR3, LPS for TLR4, FLA-ST for TLR5, CL307 for TLR7, and ODN1668 for TLR9] for 24 h. The levels of metabolites in the polyamine biosynthesis pathway were monitored using LC-MS. Our data showed that activation of TLR5, TLR7, and TLR9 increased putrescine concentration in 3D4/21 cells but decreased spermine concentration (Figure 1A). Therefore, we next examined the gene and protein expression of key enzymes involved in the polyamine biosynthesis pathway in 3D4/21 cells upon activation of TLR5, TLR7, and TLR9. The activation of these TLRs did not affect the gene expression of *ARG1*, *ODC1*, *SRM*, *SMS*, and *SMOX* (Figure 1B). However, the protein expression of ODC, SRM, and SMS was significantly decreased in TLR7- and TLR9-activated cells (Figure 1C). Collectively, these findings suggest that TLR activation inhibited the production of spermine in porcine macrophages. Interestingly, this inhibitory effect may be unique to pigs, since the TLR9 activation did not inhibit the protein expression of enzymes involved in the polyamine biosynthesis pathway in ANA.1 murine macrophages (Figure 1D).

### 3.2. TLR Activation Altered the Tryptophan Metabolism of Porcine Macrophages

**Figure 2:**
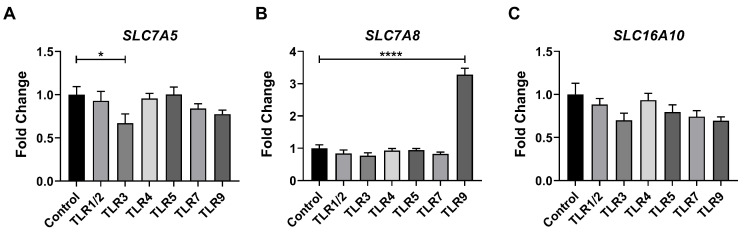
TLR activation influences the expressions of tryptophan transporters in porcine macrophages. Relative mRNA expressions of tryptophan transporters (**A**) *SLC7A5*, (**B**) *SLC7A8*, and (**C**) *SLC16A10* in control or TLR-activated macrophages. Data were analyzed by an unpaired *t* test and exhibited as the mean ± SEM. * *p* < 0.05; **** *p* < 0.0001.

To directly evaluate the potential impact of TLR activation on the tryptophan metabolism, we subsequently analyzed the expression of tryptophan transporters, specifically *SLC7A5*, *SLC7A8*, and *SLC16A10*, following TLR activation. The qPCR analysis revealed that TLR3 activation downregulated the mRNA expression of *SLC7A5,* whereas TLR9 activation upregulated *SLC7A8* expression in porcine macrophages (Figure 2A–C).

**Figure 3:**
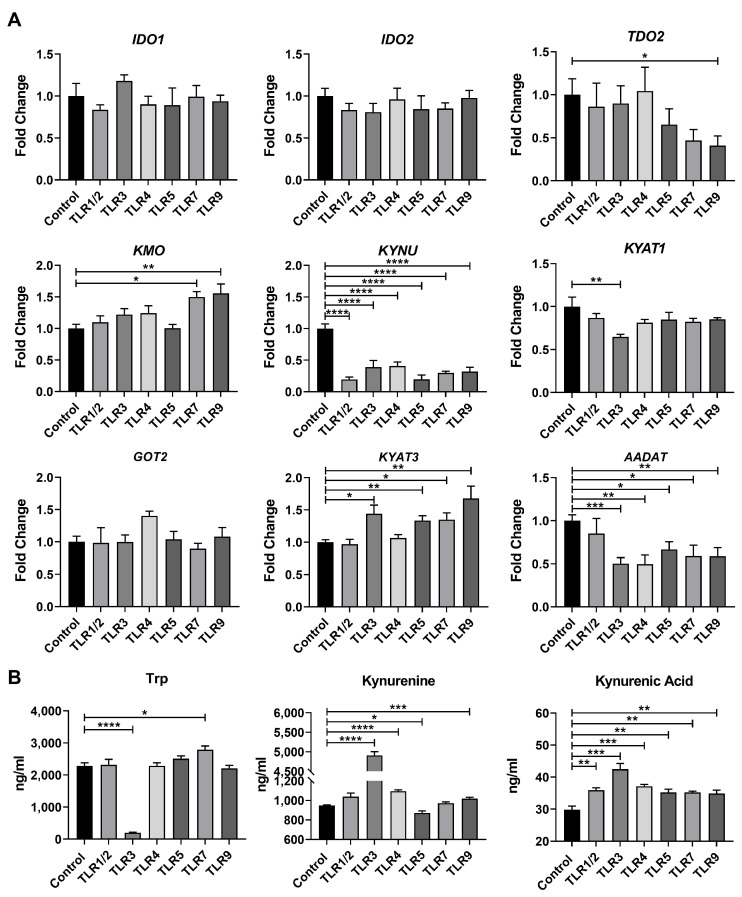
TLR activation alters the kynurenine pathway of the tryptophan metabolism toward the kynurenic acid biosynthesis. (**A**) Relative mRNA expressions of genes from the kynurenine pathway in control or TLR-activated macrophages (*n* = 5). (**B**) Levels of metabolites from the kynurenine pathway in control or TLR-activated macrophages (*n* = 6). Data were analyzed by an unpaired *t* test and exhibited as the mean ± SEM. * *p* < 0.05; ** *p* < 0.01; *** *p* < 0.001, **** *p* < 0.0001. IDO, indoleamine 2,3-dioxygenase; TDO2, tryptophan 2,3-dioxygenase 2; KMO, kynurenine 3-monooxygenase; KYNU, kynureninase; KYAT, kynurenine oxoglutarate aminotransferases; GOT2, glutamic-oxaloacetic transaminase; AADAT, aminoadipate aminotransferase; Trp, tryptophan.

The kynurenine pathway is the primary route of tryptophan catabolism. Therefore, we examined the expression of genes involved in the kynurenine pathway. Interestingly, the effects of TLR activation on the expression profiles of these genes vary depending on the specific TLR engaged (Figure 3A). Notably, TLR activation led to a significant reduction in *KYNU* expression but had no impact on the expression of genes related to kynurenine biosynthesis, except for TLR9, which decreased *TDO2* expression (Figure 3A). Additionally, the activation of TLR3, TLR5, TLR7, and TLR9 upregulated the *KYAT3* expression but downregulated the *AADAT* expression (Figure 3A). To further assess the metabolic characteristics of the kynurenine pathway following TLR activation, we employed LC-MS to quantify the concentrations of these metabolites. Unfortunately, only tryptophan, kynurenine, and kynurenic acid were detectable in the cell lysate, while the remaining metabolites in this pathway were undetected. Consistent with the qPCR results, TLR activation significantly increased kynurenic acid levels in the cell lysate (Figure 3B). Specifically, the activation of TLR3, TLR4, and TLR9 also elevated kynurenine levels (Figure 3B). Collectively, our findings indicate that TLR activation may alter the kynurenine pathway of the tryptophan metabolism, directing it toward kynurenic acid biosynthesis.

**Figure 4:**
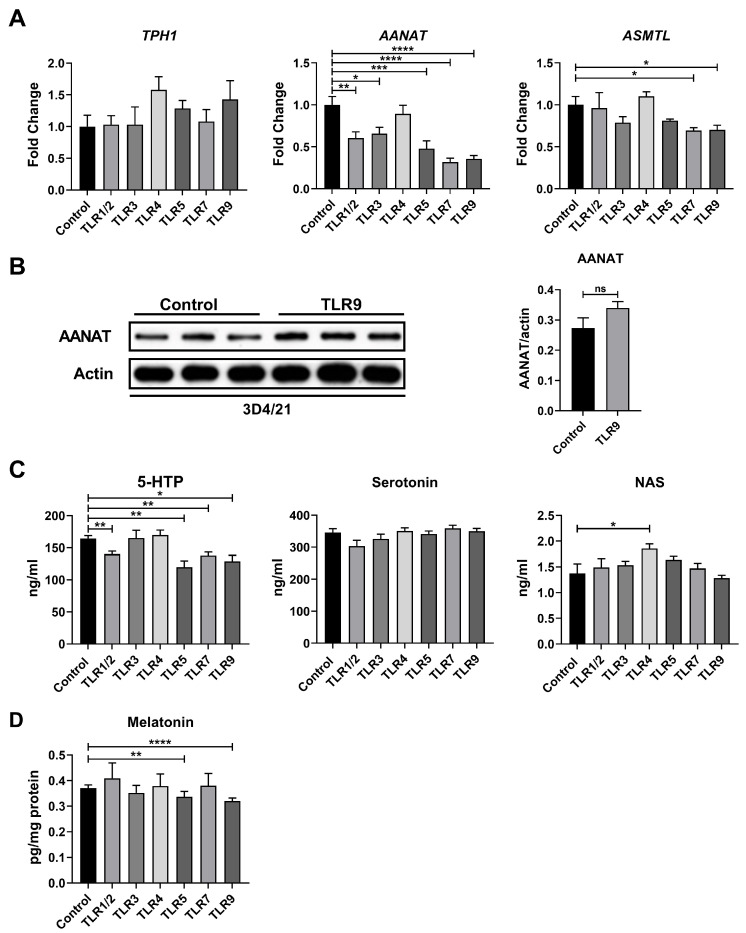
TLR activation inhibits the serotonin pathway in porcine macrophages. (**A**) Relative mRNA expressions of genes from the serotonin pathway in control or TLR-activated macrophages (*n* = 5). (**B**) Protein expressions of AANAT in control or TLR-activated 3D4/21 cells (*n* = 3). (**C**) Levels of metabolites from the serotonin pathway in control or TLR-activated macrophages (*n* = 6). (**D**) Melatonin level in control or TLR-activated macrophages (*n* = 6). Data were analyzed by an unpaired *t* test and exhibited as the mean ± SEM. * *p* < 0.05; ** *p* < 0.01; *** *p* < 0.001, **** *p* < 0.0001. 5-HTP, 5-hydroxytryptophan; NAS, *N*-acetylserotonin; TPH1, tryptophan hydroxylase 1; AANAT, arylalkylamine *N*-acetyltransferase; ASMTL, acetylserotonin *O*-methyltransferase.

In addition to the kynurenine pathway, tryptophan can be catabolized into melatonin via the serotonin pathway. Consequently, we subsequently examined gene expression within the serotonin pathway. The activation of most TLRs significantly suppressed the expression of *AANAT*, with the exception of TLR4 (Figure 4A). Furthermore, the activation of TLR7 and TLR9 also downregulated *ASMTL* expression (Figure 4A). However, we found that TLR9 activation did not affect the protein expression of AANAT (Figure 4B). To further ascertain the levels of downstream metabolites, we conducted LC-MS to quantify these metabolites. The activation of most TLRs reduced 5-hydroxytryptophan (5-HTP) levels, but had a negligible impact on the levels of serotonin and *N*-acetylserotonin (NAS) (Figure 4C). In addition to these metabolites, other relevant metabolites in the serotonin pathway remained undetected; therefore, we utilized ELISA to specifically measure melatonin levels. The activation of TLR5 and TLR9 significantly decreased melatonin levels in the cell pellet (Figure 4D). Overall, our results indicate that TLR activation inhibits the serotonin pathway in porcine macrophages.

### 3.3. The Expression of Key Genes Involved in the Tryptophan Metabolism Were Impacted by Stimulation Duration

**Figure 5:**
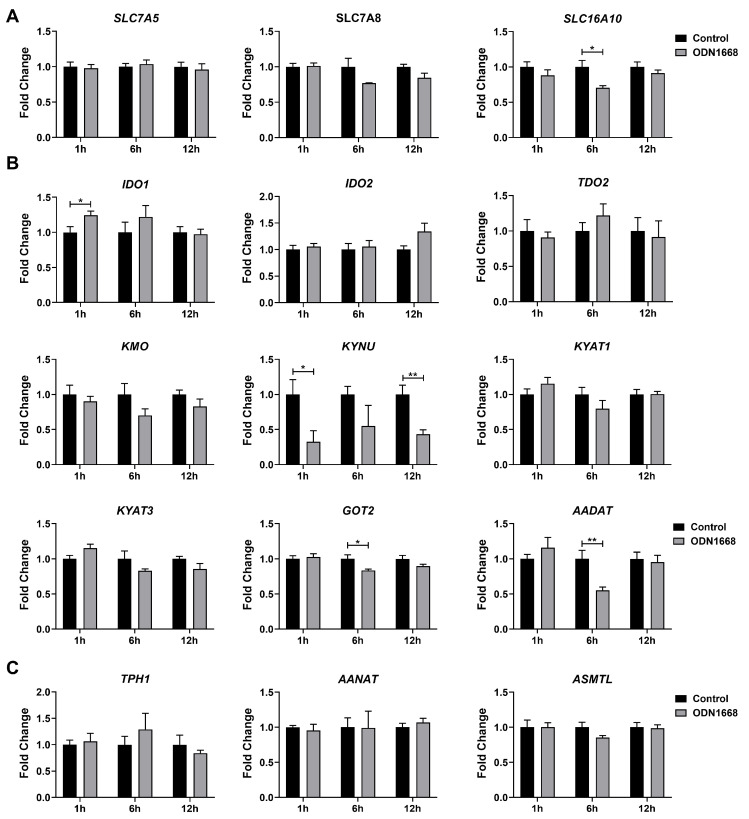
Stimulation duration of TLR9 activation influences the expression of key genes involved in the tryptophan metabolism. Relative mRNA expressions of (**A**) tryptophan transporters and metabolic enzymes in the (**B**) kynurenine pathway and (**C**) serotonin pathway in 3D4/21 cells at 1 h, 6 h, and 12 h post-TLR9 activation (*n* = 5). Data were analyzed by an unpaired *t* test and exhibited as the mean ± SEM. * *p* < 0.05; ** *p* < 0.01.

To gain insights into the dynamic impacts of TLR activation on the tryptophan metabolism, we evaluated the temporal changes in genes associated with the tryptophan metabolism at 1 h, 6 h, and 12 h after TLR9 activation. The expression of *SLC16A10* was upregulated at 6 h post-TLR9 activation, whereas other transporters remained unchanged (Figure 5A). Our analysis revealed a significant reduction in *KYNU* expression as early as 1 h after TLR9 activation, indicating suppression of the kynurenine pathway during the early stage of TLR9 activation (Figure 5B). Additionally, TLR9 activation decreased the expression of *GOT2* and *AADNT* at 6 h after stimulation (Figure 5B). In contrast, the expression of enzymes involved in the serotonin pathway showed no significant differences between TLR9-activated cells and control cells at all evaluated time points (Figure 5C). We speculated that TLR9 activation might inhibit the melatonin production only after a prolonged stimulation period of 24 h.

### 3.4. TLR9 Activation Inhibited Melatonin Production Through the PKA/cAMP/CREB Signaling Cascades

**Figure 6:**
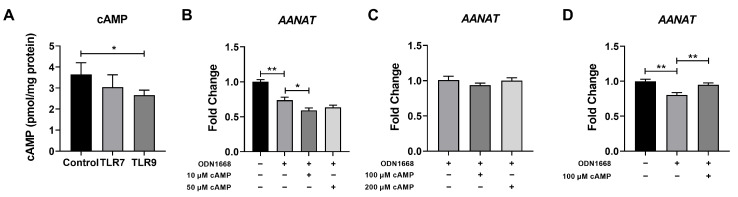
TLR9 activation inhibits melatonin production via the PKA/cAMP/CREB signaling cascades. (**A**) The cAMP level in 3D4/21 cells after TLR9 activation for 24 h (*n* = 5). (**B**,**C**) The expression of *AANAT* in control cells or TLR9-activated cells supplemented with different levels of cAMP (*n* = 5–6). (**D**) The expression of *AANAT* in TLR9-activated cells supplemented with or without 100 μM cAMP after ODN1668 clearance (*n* = 6). Data were analyzed by an unpaired *t* test and exhibited as the mean ± SEM. * *p* < 0.05; ** *p* < 0.01.

Aralkylamine N-Acetyltransferase (AANAT) serves as the pivotal rate-limiting enzyme in melatonin biosynthesis, with its expression level and enzymatic activity regulated by the PKA/cAMP/CREB signaling cascade [16,17]. Based on this, we hypothesized that TLR9 activation might inhibit melatonin production via the PKA/cAMP/CREB signaling cascade. Considering that both TLR7 and TLR9 activation suppressed the expression of *AANAT* and *ASMTL*, we measured the cAMP level in the cell pellet following 24 h of TLR7 or TLR9 activation. Consistent with previous results, TLR9 activation significantly decreased the intracellular cAMP level, whereas TLR7 activation exhibited no notable influence on the cAMP level (Figure 6A). To further validate whether the inhibitory effect of TLR9 on *AANAT* expression is mediated by the PKA/cAMP/CREB signaling cascades, we conducted an experiment where cAMP (8-Br-cAMP) was externally supplemented after TLR9 activation. Unexpectedly, cAMP supplementation failed to rescue the expression of *AANAT* (Figure 6B), even at high concentrations (Figure 6C). Upon activation, the high-affinity binding between TLRs and their ligands or adapter complexes may render the signal transduction process unidirectional and irreversible, with TLR signal transduction requiring endocytosis to remove TLR ligands or related complexes [18]. Therefore, we removed the TLR9 activator after 24 h of activation and subsequently introduced cAMP to assess *AANAT* expression after 6 h of treatment. As expected, cAMP supplementation rescued the expression of *AANAT* following TLR9 activation (Figure 6D), suggesting that TLR9 activation inhibited melatonin production through the PKA/cAMP/CREB signaling cascades.

## 4. Discussion

In pigs, a total of ten TLRs (TLR1-TLR10) have been identified [19,20]. Among these TLRs, TLR2, TLR3, TLR4, TLR5, TLR7, and TLR9 play crucial roles in regulating the immune response in pigs [21]. In this study, we observed that the activation of TLRs remodeled the polyamine and tryptophan metabolism in porcine macrophages. Specifically, we demonstrated that TLR9 activation suppressed spermine production and redirected tryptophan catabolism toward kynurenic acid biosynthesis. Furthermore, we found that TLR9 activation inhibited melatonin production via the PKA/cAMP/CREB signaling cascades. Given melatonin’s anti-inflammatory properties, its decrease could lead to the overproduction of pro-inflammatory cytokines, thereby intensifying inflammatory responses in piglets.

Polyamines play a crucial role in various cellular processes, including cell development, DNA and protein biosynthesis, antioxidation, and autophagy [22]. Notably, polyamines have been demonstrated to regulate macrophage polarization, with evidence suggesting they can inhibit polarization toward the M1 subtype while promoting the M2 subtype [23]. Our results demonstrate that the activation of TLR7 and TLR9 decreased the spermine concentration. However, activation of TLR7 or TLR9 did not downregulate the mRNA expression of related metabolic enzymes but instead decreased the protein expression of spermidine/spermine N1-acetyltransferase (SRM) and spermine synthase (SMS), suggesting that TLR7 or TLR9 activation may regulate the post-translational modifications of SRM and SMS. Spermine alleviates the pro-inflammatory response through ATG5-dependent autophagy in liver-resident macrophages [24], and thus TLR activation may promote inflammation by decreasing the intracellular spermine level.

In macrophages, melatonin influences their polarization by regulating cellular signaling pathways (e.g., NF-κB), metabolic pathways (e.g., α-KG and ROS), and miRNAs (e.g., microRNA-16b) [10,25,26,27]. Our previous research has further demonstrated that melatonin modulates macrophage function by remodeling their cellular metabolism and inhibiting the transcription of the interferon-γ receptor 2 (IFNGR2) gene, ultimately alleviating IL-1β-dependent inflammation [9]. Additionally, melatonin suppresses the production of other pro-inflammatory cytokines (e.g., TNF-α, IL-6, IL-12, and IL-10) induced by TLR9 in macrophages by inhibiting the phosphorylation of AKT within the phosphoinositide 3-kinase (PI3K) signaling pathway [28,29,30]. In the current study, our data indicate that TLR9 activation inhibits melatonin biosynthesis in porcine macrophages, potentially promoting an inflammatory response.

Melatonin production is regulated by the rate-limiting enzyme AANAT, and its expression is mediated by the PKA/cAMP/CREB signaling cascades [31]. Specifically, PKA activation enhances the phosphorylation of CREB, which then binds to the cAMP response element (CRE) in the AANAT promoter region to activate the transcription of *AANAT* [16]. We found that cAMP supplementation can eliminate the inhibitory effect of TLR9 activation on *AANAT* expression (only when the TLR9 agonist is removed), suggesting that TLR9 activation may inhibit *AANAT* expression through the PKA/cAMP/CREB signaling cascades. However, *AANAT* expression is also regulated by (Raf-1 proto-oncogene, serine/threonine kinase, RAF1)/MEK/ERK signaling cascades, which are also activated by TLR9 [17,32,33]. Additionally, miRNAs such as miRNA-483 suppress melatonin production by directly reducing *AANAT* expression [34]. Therefore, further investigation is needed to determine whether the decreased melatonin production upon TLR9 activation is modulated by other signaling pathways or miRNAs.

In summary, we have characterized the metabolic alterations in polyamine and tryptophan in porcine macrophages upon TLR activation, with a particular emphasis on TLR9. Our findings reveal that TLR9 activation not only inhibits spermine production, but also suppresses melatonin biosynthesis in the serotonin pathway via the PKA/cAMP/CREB signaling cascades. Our previous studies demonstrated that amino acids (e.g., GABA, serine, melatonin, and phenylalanine) regulate immune cell (macrophages, T cells, and B cells) fate through signaling transduction, metabolic reprogramming, epigenetic regulation, and post-translational modification [3,35,36,37,38,39]. The current research provides tailored nutritional strategies that utilize the potential of metabolites like melatonin and polyamines to regulate the immune response and mitigate diseases in piglets.

## 5. Limitations of This Study

We observed that TLR activation may regulate the kynurenine and serotonin pathways of the tryptophan metabolism by affecting gene expression. However, further investigation of the relevant metabolites in these pathways is crucial for a comprehensive assessment of the significance of these changes on our conclusions. Another weakness of this manuscript is the use of macrophage cell lines, and key results should be confirmed in primary macrophages.

## Figures and Tables

**Table 1:** List of qPCR primers used in this study.

Gene Name	Forward Primer (5′→3′)	Reverse Primer (5′→3′)
*β-actin*	GCACCGTGTTGGCGTAGAGG	GGACTTCGAGCAGGAGATGG
*SLC7A8*	TGACAACATGGAGCAGCAGCAG	AGGCAGGGAAGGAGGGAAGAAAG
*SLC7A5*	GACGCTGATGTACGCCTTCT	GCAGGCCAGGATAAAGAACA
*SLC16A10*	GCCCAATAGTCAGCGTCTTCACAG	GTCCAACAAATCCAACAGCAGCAC
*TDO2*	AAGAGCCCAGGTTCCAGGTT	CACTCACTGTTGAGCGCAGA
*IDO1*	GGTTTCGCTATTGGTGGAAA	CTTTTGCAAAGCATCCAGGT
*IDO2*	CCGTGCTCCATGCCTTTGAT	TGGCCATCTCCAGAGGACAG
*KMO*	GTTGCCCTCAGCACCACCTA	GGGAGATGCGTCCTATATTTTGG
*KYNU*	TGAGTCGCAGCTTCAACTTCATGG	ACAGGATCACGGCAATTGAGTCAC
*KYAT1*	GGTCTCCCTGTGTTTGTGTCCTTG	TTGGTGCGAGGCGTGAACTTG
*KYAT3*	CGGATGGTACGGTTGCTTGACAG	GAGACACGTCGGCGATGATGAAG
*AADAT*	CCAAGACGGTCTGTGCAAGGTG	CCATGCTCATCACTGGCAACGG
*GOT2*	AACTGGCAGCACATCGTTGACC	GGTGACCTGGTGAATGGCATGG
*TPH1*	CCGTCCTGTGGCTGGTTACTTATC	GTCCGAACCGTGTCTCACATACTG
*AANAT*	GCCACCTACCATCCCAGAGTCC	TCAAACACGCCAGCAGCATCC
*ASMTL*	TCCGCTTCCTGCCAGGTCAC	GCTTCTGCCACCAGGATGCC
*ARG1*	CTTTCTCCAAGGGTCAGC	TCCCCGTAATCTTTCACAT
*ODC1*	TGATGATTCCAAAGCAGTCTGT	AGATACATGCTGAAACCGACCT
*SRM*	CCCTCGCAAGGTGCTGAT	AGTAGCTGACAGCCATGC
*SMS*	GGCAAAGAAGTTGACAGTCTTTTGA	GGCCATCGGCAGTGGG
*SMOX*	CGGCAGTTCACGGGGAAT	CCGAGAACAGCACCTGCAT

## Data Availability

The original contributions presented in this study are included in the article. Further inquiries can be directed to the corresponding author(s).
